# A new subspecies of *Nitokra affinis* Gurney, 1927 (Copepoda, Harpacticoida) from the Caribbean coast of Colombia

**DOI:** 10.3897/zookeys.378.6695

**Published:** 2014-02-06

**Authors:** Juan M. Fuentes-Reinés, Eduardo Suárez-Morales

**Affiliations:** 1 Universidad del Magdalena, Grupo de Investigación en Limnología. A.A 731 Santa Marta, Magdalena, Colombia; 2 El Colegio de la Frontera Sur (ECOSUR), A.P. 424, 77014 Chetumal, Quintana Roo, Mexico

**Keywords:** Harpacticoids, taxonomy, meiofauna, marine crustaceans, lagoon systems biota

## Abstract

Plankton samples from Laguna Navio Quebrado, La Guajira, northern Colombia, yielded male and female specimens of an harpacticoid copepod that was first identified as the widely distributed species *Nitokra affinis* Gurney, 1927 for which at least four subspecies have been described from different geographic areas. A more detailed examination of the Colombian specimens revealed that it differs from the other morphs so far considered as subspecies. The Colombian specimens differ from the other four known subspecies in the following aspects: (1) rostrum with long projection, (2) relatively long exopod of P1, almost as long as first endopodal segment, (3) endopodal and exopodal rami of P2 equally long, (4) a reduced number of endopodal setal elements of the male P5. It also differs from *N. a. californica* Lang in details of the ornamentation of the urosomites. Descriptions and illustrations of this new subspecies, the first one described from the Neotropical region, are presented together with a key to the five known subspecies of *Nitokra affinis*. As in many other cases of presumedly widespread species of harpacticoids, it is possible that *N. affinis* represents a species complex with more restricted distributional patterns, a notion that certainly deserves further study.

## Introduction

The family Ameiridae is one of the most diverse among the harpacticoids; the genus *Nitokra* Boeck, 1865 is contained in this group. Species of this genus occur in fresh, brackish and marine water habitats (Karanovic and Pesce 2002), as well as a wide range of sediment types (Boxshall and Halsey 2004). *Nitokra* is considered a diverse taxon which is currently known to contain over 45 valid species (Wells 2007; Gómez et al. 2012), some of them with a remarkable morphologic variability that has motivated the erection of subspecific taxa. Currently, eight species (*Nitokra affinis* Gurney, 1927, *Nitokra divaricata* Chappuis, 1923, *Nitokra fallaciosa* Klie, 1937, *Nitokra hibernica* (Brady, 1880), *Nitokra lacustris* (Shmankevich, 1875), *Nitokra mediterranea* Brian, 1928, *Nitokra minor* Willey, 1930, *Nitokra platypus* Daday, 1906) are known to contain 22 subspecies (Wells 2007). *Nitokra affinis* is a widespread species containing four subspecies: *Nitokra affinis affinis* Gurney, 1927, *Nitokra affinis rijekana* Petkovski, 1954, *Nitokra affinis californica* Lang, 1965, and *Nitokra affinis stygia* Por, 1968 (Wells 2007).

In Colombia only three species and subspecies of *Nitokra*: the first record, involving the description of the subspecies *Nitokra lacustris pacifica* Reid, 1987 was published by Reid (1987). More recently *Nitokra lacustris sinoi* Por & Marcus, 1976 and *Nitokra taylori* Gómez, Carrasco & Morales-Serna, 2012 (Fuentes-Reinés and Suárez-Morales in press) were added to the national records of the genus.

From a biological survey of a coastal lagoon system in the Caribbean coast of Colombia, specimens of a species tentatively identified as *Nitokra affinis* Gurney, 1927 were obtained; a further analysis of these specimens revealed that they show consistent differences with respect to the other four subspecific forms currently known. In this contribution we describe and illustrate this taxon and provide a key to the identification of the five subspecies of *Nitokra affinis*.

## Materials and methods

Samples of near-shore and open water plankton were taken from the Laguna Navío Quebrado, Colombia (11°25'N, 73°5'W) from April to December 2012. Samples were mainly in the littoral areas with vegetation (macrophytes and mangrove) but also from limnetic areas close to oyster banks. Water salinity ranged from 0 to 28‰. Water samples were collected using a bucket of 25 L at both littoral vegetation areas and open water. Samples were filtered with a standard zooplankton net with a 45 μm mesh and fixed and preserved in 70% ethanol. Dissected specimens and appendages were mounted in glycerine and sealed with Canada balsam. Drawings of the mounted appendages were prepared with a camera lucida and also photographed using a Kodak Easy Share C140 digital camera adapted to a compound microscope. The specimens were measured in lateral position, from the tip of rostrum to the posterior margin of the caudal rami. Morphological nomenclature follows the terminology proposed by Huys and Boxshall (1991). The following abbreviations are used in the text and tables: P1–P6, first to sixth swimming legs; EXP, exopod; ENP, endopod. The type specimens examined were deposited in the collection held at the Museo de Colecciones Biológicas de la Universidad del Atlántico (UARC), Barranquilla, Colombia. Additional specimens were deposited in the collection of zooplankton held at El Colegio de la Frontera Sur, Chetumal, Mexico (ECO-CHZ).

## Results

### Family Ameiridae Boeck, 1865
Subfamily Ameirinae Boeck, 1865
Genus *Nitokra* Boeck, 1865

#### Nitokra affinis colombiensis ssp. n.

http://zoobank.org/B6610CBE-C007-4B2A-A2B6-3EE5ADF4BDD6

http://species-id.net/wiki/Nitokra_affinis_colombiensis

##### Material examined.

One female holotype (UARC133M) and one male allotype (UARC134M), ethanol-preserved. Paratypes: one female (UARC142M-147M) and one male (UARC136M-141M), plus two females and two males (UARC135M). Additional material: Six adult females, four adult males in authors’ (JF-R) personal collection. Two adult females, two adult males from same locality and date (ECO-CHZ-09088).

##### Type locality.

Laguna Navío Quebrado, La Guajira, Colombia (11°25'N, 73°5'W).

##### Female.

**Figure 1.**
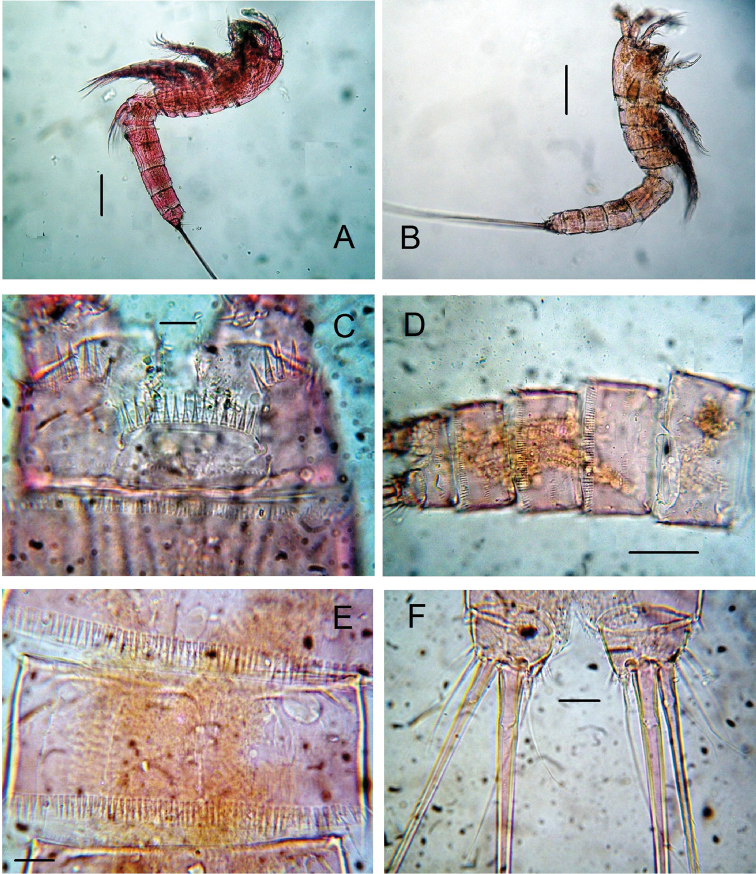
*Nitokra affinis colombiensis* ssp. n., from northern Colombia. **A** adult female, habitus, lateral view **B** adult male, habitus, lateral view **C** female, anal somite showing ornamentation of anal operculum **D** male, urosome, ventral view **E** male, third urosomite, ventral view **F** male, caudal rami, ventral view. Scale bars: **A, B** = 100 μm, **C, E, F** = 10 μm, **D** = 50 μm.

**Figure 2.**
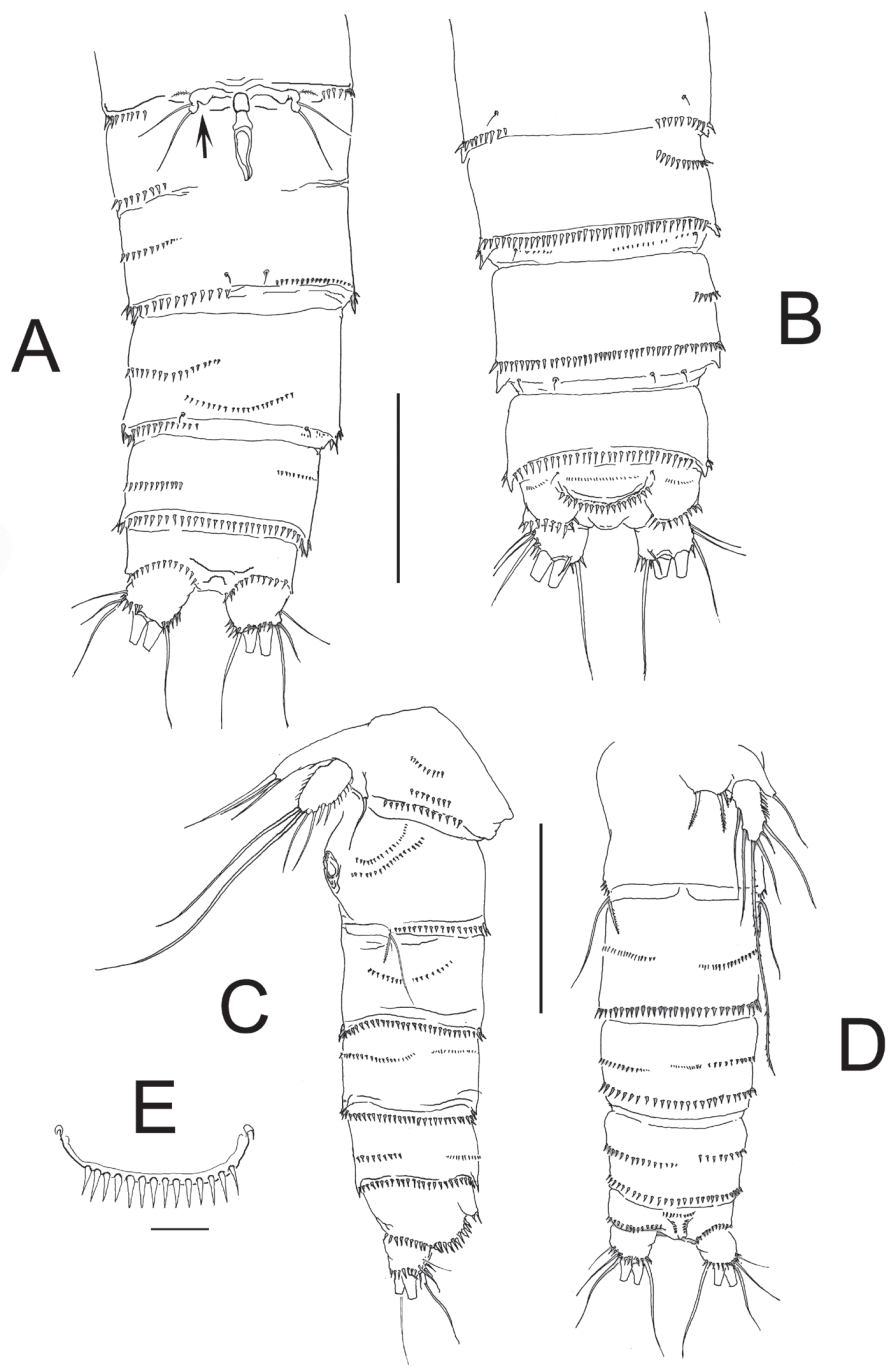
*Nitokra affinis colombiensis* ssp. n., from northern Colombia. **A** female, urosome, ventral view showing genital field and P6 **B** same, dorsal view, showing genital field and sixth leg plate, arrowed **C** male, urosome, lateral view showing P5 and P6 plate **D** same, ventral view **E** male, detail of ornamentation of anal operculum. Scale bars: **A–D** = 100 μm, **E** = 10 μm.

**Figure 3.**
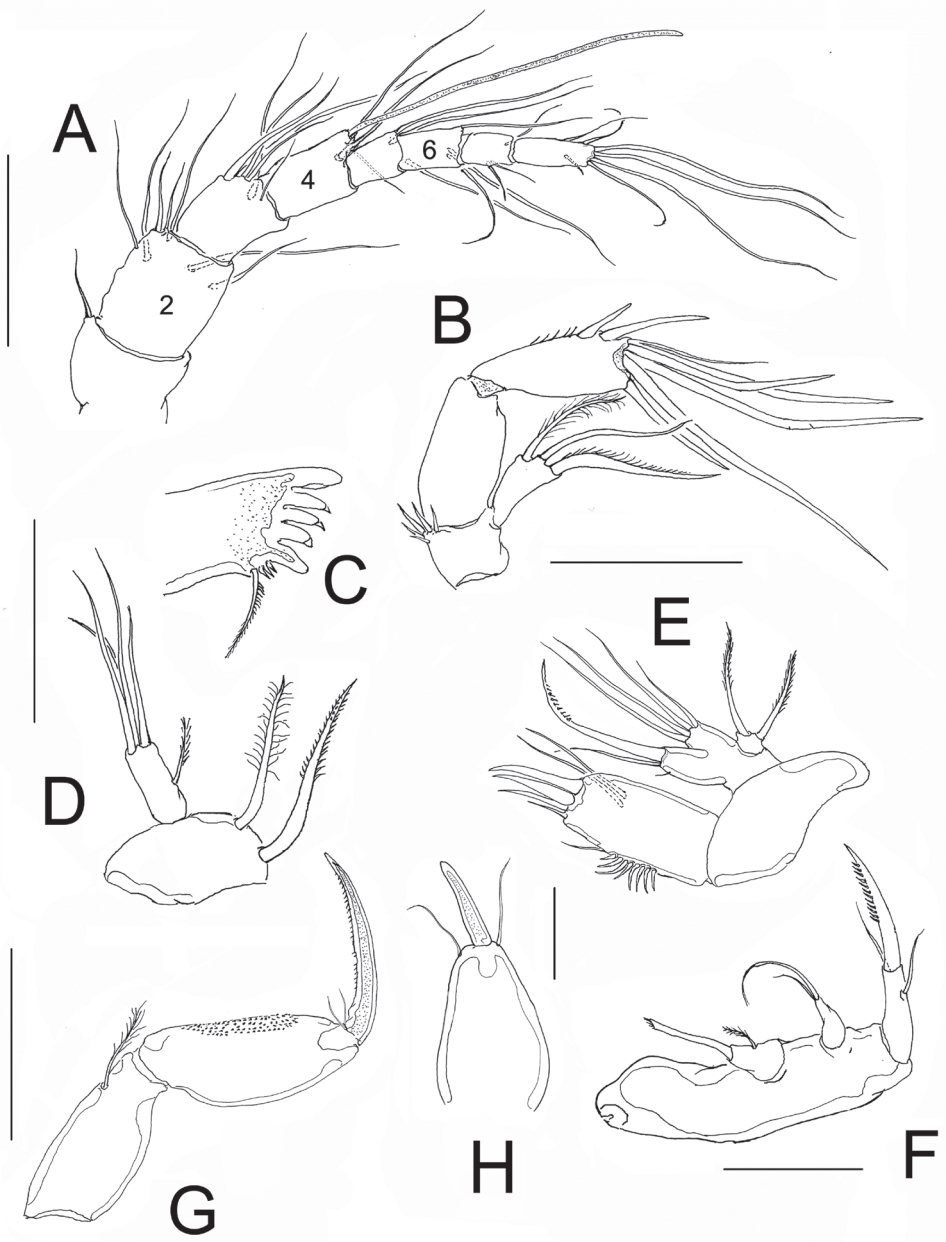
*Nitokra affinis colombiensis* ssp. n., adult female from northern Colombia. **A** antennule **B** antenna **C** mandible blade **D** mandibular palp **E** maxillule **F** maxilla **G** maxilliped **H** rostrum with rostral process. Scale bars: **A–G** = 50 μm, H = 10 μm.

Habitus in lateral view as in Figure 1A. Body subcylindrical, tapering posteriorly, total body length measured from tip of rostrum to posterior margin of caudal rami ranging from 588 to 714 μm (average 660 μm, *n* = 6; holotype: 700 μm). Rostrum subtriangular with 2 sensilla and apical rostral projection which is about half the length of rostrum (Fig. 3H). Genital double-somite distinct dorsally and laterally, with partial ventral suture (Fig. 2A, B). Anterior ventral surface of genital somite with incomplete rows of spinules on medial outer surface, distal row of spinules and pair of sensilla. Succeeding urosomite with dorsal incomplete row of spinules on medial surface and distal row of spinules covering lateral margin and only part of ventral margin (Fig. 2A, B); same somite with ventral curved row of minute spinules on central surface, incomplete row on medial outer margin and 2 sensilla on posterior margin. Preanal somite with similar ornamentation pattern except for spinules encircling posterior margin of somite, absence of curved row on ventral surface and additional row of minute spinules. Anal somite with ventral and dorsal rows of spinules along posterior margin bordering insertion of caudal rami; anal operculum semicircular, ornamented with 14–20 large spinules, flanked by 2 sensilla and rows of minute spinules (Figs 1C, 2B). Caudal ramus short, subquadrate, with rounded margins, armed with 6 setae, seta I small, seta II as long as seta I, seta III on distal outer position, about 1.5 times as long as setae I and II. Setae IV and V thick, long, the former being longest; seta VI slightly longer than seta III. Dorsal seta VII uniarticulate at base. Ramus ornamented with spinules at insertion of setae (Figs 1F, 2A, B).

*Antennule*. 8-segmented, second segment about 1.5 longer than first and third segments, aesthetasc on fourth segment reaching well beyond distal end of terminal segment (Fig. 3A). Second segment longest, about 1.5 times as long as third segment and 2.1 times longer than preceding first segment. Fourth segment about as long as third segment. Armature as follows: 1(1), 2(7), 3(7), 4(3+aes), 5(2), 6(3), 7(3), 8(7).

*Antenna*. Basis with group of 5 unequal spiniform setae, first endopodal segment subrectangular, smooth, second endopodal segment with subdistal row of spinules on inner margin, with 2 lateral inner spines and 6 distal elements, outermost two of them basally fused at insertion. Exopod one-segmented with 3 setae, 2 pinnate and 1 smooth seta (Fig. 3B).

*Mandible*. Gnathobase with 5 large teeth, and long dorsal seta ornamented with short spinules (Fig. 3C). Mandibular palp 2-segmented, first segment (basis) with 2 setae. Endopodal segment with 1 short lateral and 4 long apical setae (Fig. 3D).

*Maxillule*. Arthrite of praecoxa ornamented with group of subequal spinules, arthrite armed with 2 subdistal setae and 4 distal elements. Coxal endite with 2 setae. Basis with 4 setae; exopod 1-segmented, with 2 setae, one slender, the other thicker, distally serrate (Fig. 3E).

*Maxilla*. Syncoxa naked, with 2 endites, proximalmost with single, slender modified element with distal tuft of setules and with short proximal seta; second endite with 2 unequal setae. Allobasis forming strong serrate claw with 2 accessory setae on proximal position (Fig. 3F).

*Maxilliped*. Subchelate. Syncoxa with single seta on inner distal corner, basis unarmed, with longitudinal patch of spinules. Endopod drawn into long and slender lightly serrate claw with 2 short accessory setae (Fig. 3G).

**Figure 4.**
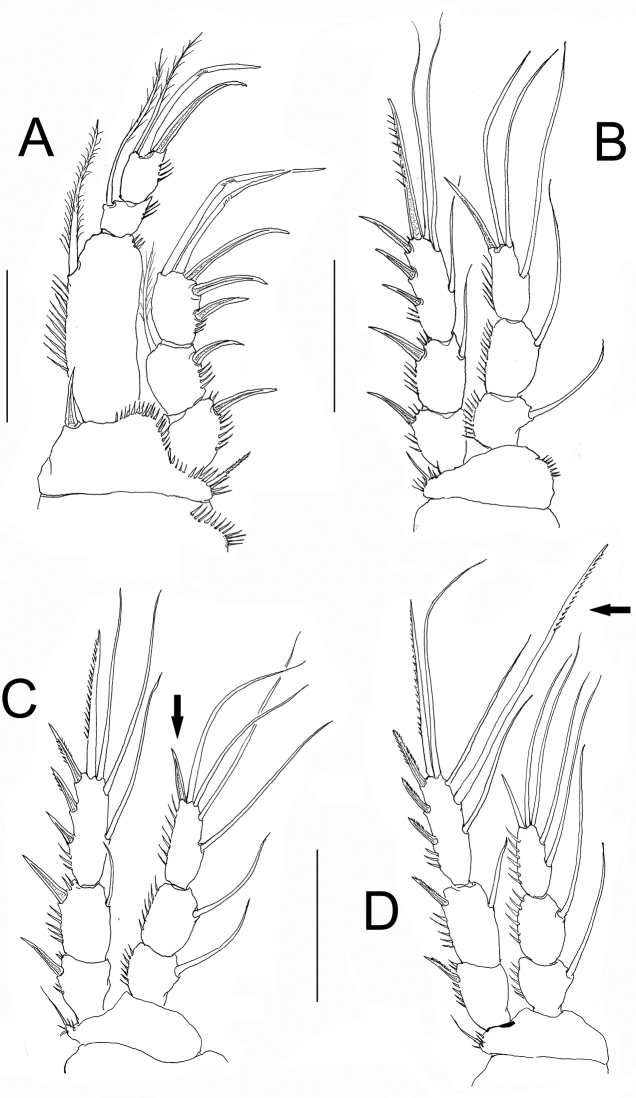
*Nitokra affinis colombiensis* ssp. n., adult female from northern Colombia. **A** first swimming leg (P1) **B** second swimming leg (P2) **C** third swimming leg (P3) **D** fourth swimming leg (P4) showing modified pectinate element (arrowed). Scale bars: **A–D**= 50 μm.

*P1*. Coxa with outer row of slender spinules. Basis with spinules bordering insertion of exopodal and endopodal rami, inner basipodal spine short, stout, reaching 1/3 of length of first endopodal segment. Outer basipodal spine short, stout, spinulated. EXP and ENP 3-segmented. Exopodal ramus shorter than first endopodal segment. Third exopodal segment with 2 apical geniculate setae. First endopodal segment about 2.9 longer than its width; third endopodal segment with terminal claw, the latter about 1.5 times as long as segment (Fig. 4A).

*P2*. Coxa with outer row of slender spinules. Basipod with 2 groups of spinules, as figured. Exopod and endopod 3-segmented. Exopod endopod equally long. First exopodal segment without inner seta, second and third exopodal segments with inner seta. Exopod without modified setae, outer margin of exopodal segments spinulated. Endopod 3-segmented, outer margin of segments ornamented with spinules (Fig. 4B).

*P3*. Coxa with outer row of slender spinules. Basipod with spinules only at insertion of outer basipodal seta. Exopodal and endopodal rami as in P2 except for shorter apical spiniform element (arrowed in Fig. 4C) and endopod slightly shorter than exopod (Fig. 4C).

*P4*. Coxa with outer row of slender spinules. Basipod as in P3. EXP longer than ENP (Fig. 4D). Middle inner seta of EXP3 thicker and longer than adjacent setae (arrowed in Fig. 4D).

*P5*. EXP subrectangular, about 1.66 longer than it is width, with 6 setae. Relative length of exopodal setae from inner to outer element as follows: 0.81, 1.00, 0.18, 0.38, 0.18, 0.56. Endopodal lobe quadrate, reaching almost halflength of EXP, with 5 spinulose setae, outermost being longest; relative length of setae from inner to outer elements as follows: 0.32, 0.37; 0.43, 1, 0.46 (Fig. 6A).

Armature formula of female P1-P5 as follows:

**Table:** 

	P1	P2	P3	P4	P5
EXP	I-0;I-1;III,2,0	I-0;I-1;III,I,1,2	I-0;I-1;III,I,1,2	I-0;I-1;III,I,1,3	6
ENP	0-1; 0-1;I,2,0	0-1;0-1;I,2,1	0-1;0-1;I,2,2	0-1;0-1;I,2,2	5

*P6*. Represented by narrow plate with subdistal lobe-like process marked by a notch (arrowed in Fig. 2A); plate bearing 3 elements, 2 equal slender setae and outer small spinulated seta (Fig. 6B).

##### Male.

Smaller than female, habitus in lateral view as in figure 1B. Total body length ranging from 518 to 574 μm (mean, 546 μm; *n* = 4; allotype: 518 μm). Ornamentation of urosomites resembling that of female except for position of rows of minute spinules on ventral surface of genital and preanal somites (Fig. 2C, D). Anal somite with row of small spinules on posterior margin at insertion of caudal rami. (Figs 1D, E, 2C, D). Rostrum, antennae and mouthparts as in female.

**Figure 5.**
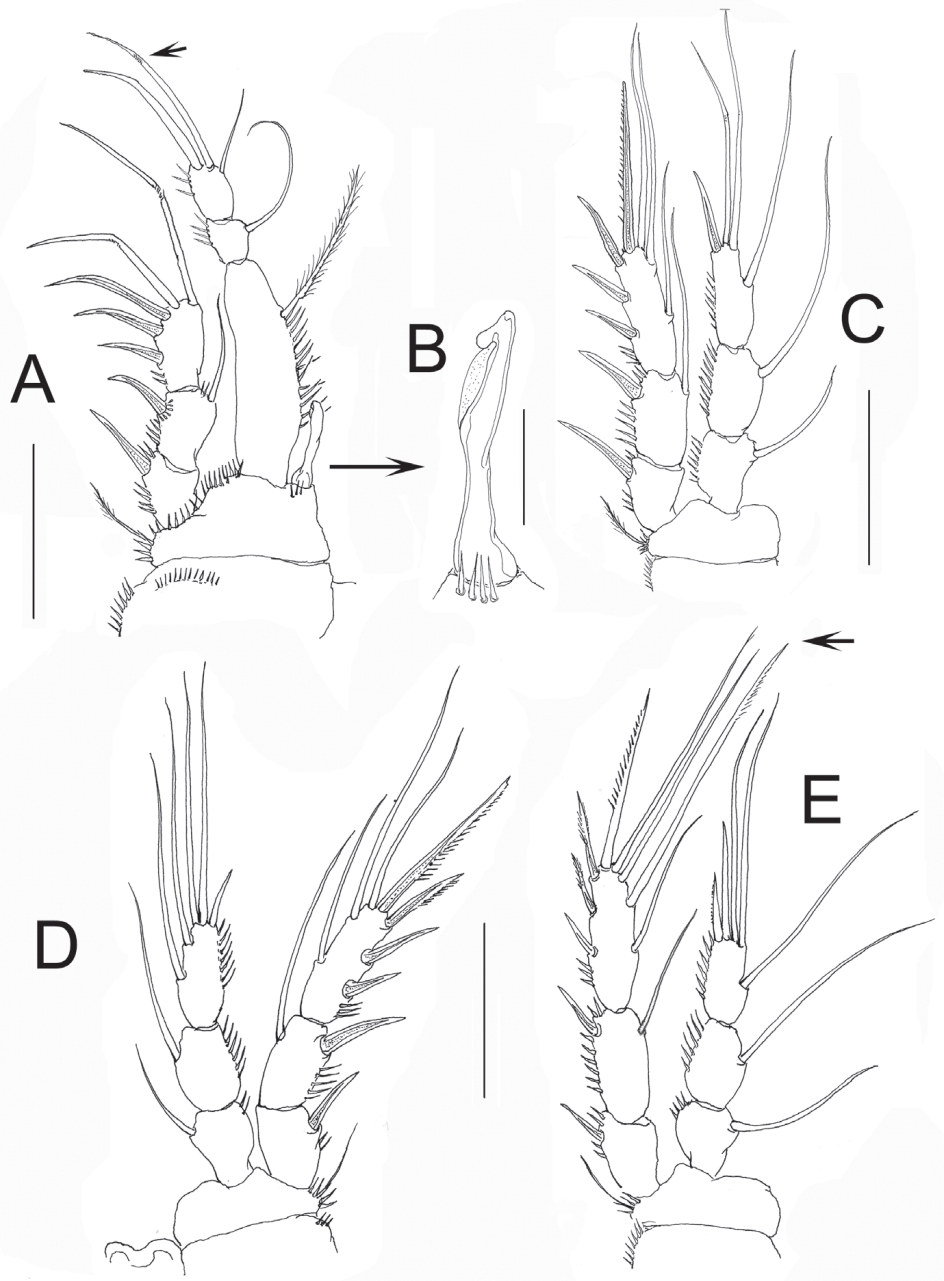
*Nitokra affinis colombiensis* ssp. n., adult male from northern Colombia. **A** first swimming leg (P1) **B** modified inner basipodal spine of P1 **C** second swimming leg (P2) **D** third swimming leg (P3) **E** fourth swimming leg (P4). Scale bars: **A, C–E** = 50 μm, **B** = 10 μm.

*P1*. As in female except for an additional row of spinules on the coxa, presence of small slender seta and 1 geniculate apical seta (arrowed in Fig. 5A), instead of 2 on ENP3, slenderer ENP1, and dimorphic modified inner basipodal spine (Fig. 5A, B).

*P2-P4*. As in female (Fig. 5C-E), including thickened middle inner seta of EXP3 (arrowed in Fig. 5E) which is relatively shorter than in female.

**Figure 6.**
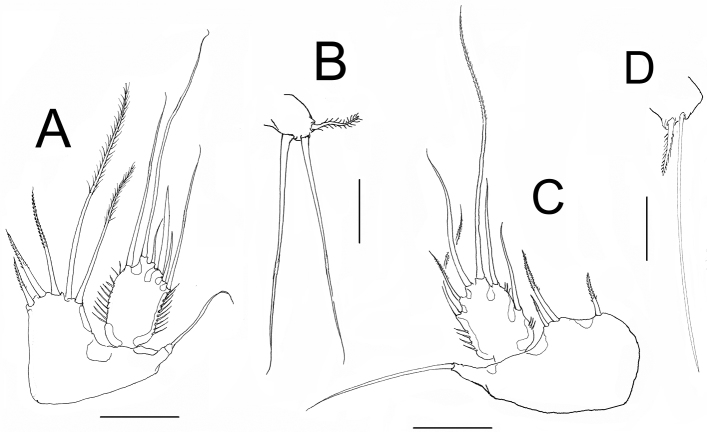
*Nitokra affinis colombiensis* ssp. n. from northern Colombia. Adult female: **A** fifth leg **B** sixth leg. Male: **C** fifth leg **D** sixth leg. Scale bars: **A, C** = 50 μm; **B, D** = 10 μm.

*P5*. EXP subquadrate, armed with 6 setae, distal innermost being longest, reaching midlength of fourth urosomite (Fig. 2C, D). Baseoendopod with 3 unequally long setae, middle one longest, about twice as long as the other two (Fig. 6C).

*P6*. With 2 unequal seta, inner one three times longer than outer seta (Figure 6D). Caudal rami as in female (Fig. 2C, D).

Variability. One male with 2 elements (instead of3) on ENP3 of P1.

##### Etymology.

The new subspecies is named after Colombia, the country from which it was first discovered.

##### Habitat.

The estuary Laguna Navío Quebrado has a surface area of 10.7 km^2^; it is characterized by the presence of an oyster bank in the limnetic area and vegetation (mangrove and beds of macrophytes) in the littoral zone. Water temperature ranged between 28 and 31 °C, salinity between 0-28 psu, and pH values were 7.8–8.3.

## Discussion

Based on the combination of the armature formula of the P1EXP2-3, three species groups can be recognized within the genus *Nitokra* (Gómez et al., 2012). The first group exhibits one inner seta and four elements on P1EXP2 and EXP3, respectively, the second group lacks an inner seta on P1EXP2 but bears five setae on P1EXP3. The third group exhibits one inner seta, and five setal elements on P1EXP2 and EXP3, respectively. *Nitokra affinis* Gurney is part of this third group. This is a very widespread species, recorded from different geographic regions, including the Atlantic and Pacific Oceans, the Mediterranean, the Black Sea, the Red Sea, the Caribbean, and Brazil (Gurney 1927; Vervoort 1962, 1964; Lang 1965; Por et al. 1984; Suárez-Morales et al. 2006). According to Vervoort (1964) most records of *Nitokra affinis affinis* are related to sandy sediments including interstitial water of beaches but at least two subspecies have been collected from cave-related environments (Por 1968). Our specimens from Colombia were collected in open water and mangrove areas.

Despite the fact that Lang (1965) expressly used the term “forma” in his description of *Nitokra affinis californica* and thus caused the nomen to be infrasubspecific according to the ICZN (art. 45.6); the subspecies rank is reinstated when Por (1968) proposed this rank for the other formae described in the group, thus meeting the requirements stated by the ICZN (art. 45.6.4.1). Hence, these forms should be recognized as subspecies.

The four known subspecific forms of *Nitokra affinis* are known from different geographic areas: *Nitokra affinis affinis* from the Suez Canal, European and Mediterranean waters, and Bermuda (Gurney 1927; Willey 1930; Chappuis 1938; Roe 1958; Vervoort 1962); *Nitokra affinis stygia* Por, 1962 from the Red Sea; *Nitokra affinis californica* from Monterey Bay, California, and *Nitokra affinis rijekana* from Yugoslavia and Tenerife (Petkovski 1954; Noodt 1958). There are two additional records of *Nitokra affinis californica*, one from South Africa (Kunz 1975) and the other one from Bulgaria (Apostolov 1980). It is likely that the Bulgarian and the South African specimens might represent different subspecies but the available morphological data are insufficient to advance a conclusive statement. This notion is supported by the presence of a clearly shorter P1 exopod in both the Bulgarian and South African material, the exopodal ramus reaches only about ¾ of the length of the first endopodal segment, clearly diverging from the equally long exopod and first endopod segment condition that is diagnostic of *Nitokra affinis californica* (Lang, 1965). In addition, the relative lengths of the setae of the male fifth leg and the length/width proportions of the female exopodal segment show some differences with respect to Lang’s (1965) *Nitokra affinis californica* (see Kunz 1975, table I; Apostolov 1980, Figs 1e, f). The number of subspecies of *Nitokra affinis* could be underestimated.

The Colombian specimen shares most characters with *Nitokra affinis* Gurney, and its subspecific forms, including the armature formula of P1-P4, the morphology of the mouthparts, the size proportions and armature of the caudal rami, and the number of setae on the female and male P5EXP. The new subspecies, *Nitokra affinis colombiensis* ssp. n. differs from its congeners in the following aspects: (1) in the Colombian specimens the rostrum has a long rostral projection. This structure has not been hitherto described or depicted in any other subspecies of *Nitokra affinis*; (2) the length of the EXP with respect to the enlarged P1ENP1 differs among these subspecies; in *Nitokra affinis affinis* and *Nitokra affinis rijekana* the exopod reaches about the point of insertion of the inner seta of the first endopodal segment (Gurney 1927; Lang 1965), whereas in *Nitokra affinis stygia* the exopod is clearly shorter and does not reach this level (Por 1968). In *Nitokra affinis californica* the exopod is longer, it reaches well beyond this point and it is about as long as the endopodal segment (Lang 1965). In the new subspecies the EXP reaches beyond the insertion of the inner endopodal seta but is shorter than the first endopodal segment; (3) in the new subspecies *Nitokra affinis colombiensis* the endopodal ramus of P2 reaches the distal margin of the exopod. In the other known subspecies the endopod does not reach beyond half the length of the third exopodal segment (Lang 1965), (4) the new subspecies can be readily distinguished by the number of elements of the male P5 baseoendopod, it has three setae vs. 5 in *Nitokra affinis rijekana*, 4-5 in *Nitokra affinis affinis*, and 4 in *Nitokra affinis californica* and *Nitokra affinis stygia* (Petkovski 1954; Lang 1965; Por 1968) and (5) in *Nitokra affinis colombiensis* the ornamentation of the posterior margin of the postgenital somite is similar to the strict form of *Nitokra affinis*, with spinules absent on the ventral margin, but differs from the pattern described in both *Nitokra affinis rijekana* and *Nitokra californica* in which the somite is encircled by spinules (Lang 1965). It also diverges from *Nitokra affinis stygia*, with a naked dorsal margin (Por 1968, pl. 5, fig. 28).

Overall, the new subspecies most closely resembles *Nitokra affinis californica*, but some additional characters can be useful to separate these two species; the number of spines on the posterior margin of the female anal operculum is only 14-20 in the new subspecies (Fig. 2H) vs. +25 in *Nitokra affinis californica* (Lang, 1965, fig. 196b). The second antennular segment of *Nitokra affinis californica* is relatively longer (1.7 times as long as third segment) than in *Nitokra affinis colombiensis* (1.3). Also, the fourth segment is elongate in *Nitokra affinis californica* (3.3 times as long as wide) and clearly shorter (1.4) in the new subspecies. The ornamentation of the maxillipedal basis is represented by row of short hair-like elements in *Nitokra affinis californica* (Lang, 1965, fig. 197c) whereas this segment has a patch of spinules in the Colombian specimens. The shape and armature of the female sixth leg plate differs between these taxa, the two inner setae are unequally long in *Nitokra affinis californica* but these elements have the same length in the Colombian specimens (Figs 2A, 6B). Also, in the new subspecies the distal section of the plate has a subterminal notch (arrowed in Fig. 2A) which is absent in *Nitokra affinis californica* (Lang, 1965, fig. 196d). The shape of the male fifth leg exopod is clearly subrectangular in the new subspecies *vs.* subtriangular in *Nitokra affinis californica* (Lang, 1965, fig. 197 h). In addition, the middle apical seta of the male fifth leg exopod is distinctively long in the Colombian specimens, it reaches midlength of the fourth urosomite (Fig. 2C, D) whereas this seta is clearly shorter in the Californian subspecies, barely reaching beyond the second urosomite (Lang 1965, fig. 197f). The ornamentation of the male urosome is different in these two forms; *Nitokra affinis californica* has a more complex ornamentation pattern on the lateral surface of the second and third urosomites, with 5 and 7 transverse rows of spinules, respectively (Lang 1965, fig. 197f) *vs.* a clearly lighter ornamentation in the Colombian form (1 and 2 rows, respectively).

As in many other cases of presumedly widespread species of harpacticoids, it is possible that *Nitokra affinis* represents a species complex with more restricted distributional patterns, a notion already advanced by Vervoort (1964). The status of subspecific taxa in the genus *Nitokra* has been modified to recognize independent species on the basis of consistent morphological differences (Gómez et al. 2012). The comparative morphological data provided by Lang (1965) about *Nitokra affinis* and the additional characters explored in this work appear to be a sound frame to define species boundaries for use in taxonomic discrimination in this species complex. The lack of detail in the original description of most of these subspecific taxa prevents a full comparative examination of characters leading to advance further in this direction. In addition, it has to be considered that the known morphological variability of the group together with the morphological stasis and convergent evolution of character states could hinder this task (Easton et al. 2010). Gene-sequencing studies have been proved to be a useful tool for species delimitation among harpacticoids (Rocha-Olivares et al. 2001); hence, if morphological differences are deemed uninformative, these techniques are the next step to take in testing the validity of these five subspecific taxa of *Nitokra affinis* at the species rank. The use of the generic name *Nitokra* instead of *Nitocra* follows Bowman (1988) and Walter and Huys (2013). The former nomen is the original spelling and despite its widespread use, *Nitocra* has not been officially validated.

### Key to the subspecies of *Nitokra affinis* Gurney, 1927

**Table:** 

1A	Female P1ENP1 less than 3.8 times as long as wide; EXP of P1 reaching the point insertion of inner seta of ENP1 (Figs 3A, 4A); male P5ENP with 3 setae; rostrum with rostral projection	*Nitokra affinis colombiensis* ssp. n.
1B	Female P1ENP1 more than 3.8 times as long as wide; EXP of P1 with a different length; male P5ENP with 4 or 5 setae; rostrum without rostral projection	2
2A	Middle inner seta of P4EXP3 (arrow in Fig. 3D) not longer and stronger than distal inner seta; male P5ENP with 5 setae	*Nitokra affinis rijekana* Petkovski, 1954
2B	Middle inner seta of P4EXP3 longer and stronger than the distal inner seta	3
3A	Posterior edge of antepenultimate somite with incomplete spinules ring, dorsal to ventro-lateral only, ventral margin smooth	*Nitokra affinis affinis* Gurney, 1927
3B	Posterior margin of antepenultimate somite spinulose as a continuous ring	4
4A	P1EXP short, not reaching insertion of inner seta of P1 ENP1; female P5EXP not elongated at distal half, about 1.3 as long as wide, innermost distal seta about as long as adjacent distal seta	*Nitokra affinis stygia* Por, 1962
4B	P1EXP long, reaching well beyond insertion of inner seta of P1ENP1, both rami equal in length; P5EXP elongated at distal half, about 1.5–1.8 as long as wide; innermost distal seta about 1.5 times as long as adjacent distal seta	*Nitokra affinis californica* Lang, 1965

## Supplementary Material

XML Treatment for Nitokra affinis colombiensis
